# Therapeutic Effects of Taurine and Histidine Supplementation in Retinal Diseases

**DOI:** 10.3390/life14121566

**Published:** 2024-11-29

**Authors:** Deokho Lee, Lois E. H. Smith

**Affiliations:** Department of Ophthalmology, Boston Children’s Hospital, Harvard Medical School, Boston, MA 02115, USA; deokho.lee@childrens.harvard.edu

**Keywords:** retinal metabolism, retinal diseases, amino acids, nutrients, taurine, oxidative stress, angiogenesis

## Abstract

Amino acids are basic building blocks of structural proteins and enzymes. They also act as signaling molecules and as fuel. They are characterized as essential if sufficient quantities must be supplied exogenously or as non-essential if they can be endogenously synthesized. Appropriate intake of amino acids not only prevents the development of metabolic diseases but also can reduce the progression of some disease states. Amino acids are strongly associated with retinal metabolism in physiology and pathology. Nonetheless, there is a lack of robust clinical studies supporting the benefits of amino acid supplementation in retinopathy. In this review, we summarize preclinical evidence concerning the potential of supplementing the amino acids taurine and histidine to provide protection against diabetic retinopathy, glaucoma, and age-related macular degeneration. We suggest further directions for studying amino acid-based therapeutic interventions for eye diseases.

## 1. Introduction

**Figure 1:**
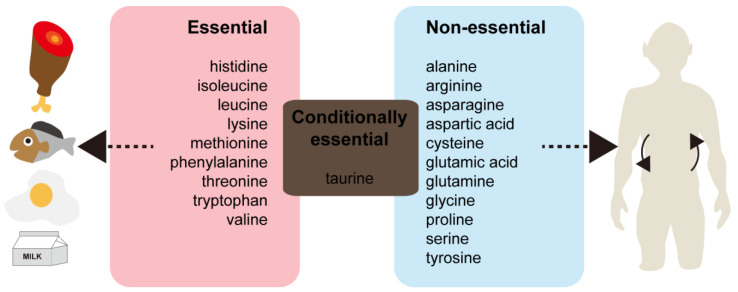
Amino acids are divided into essential and non-essential groups. Amino acids are organic compounds containing amino and carboxylic acid functional groups. Approximately 500 amino acids have been identified in nature. Essential amino acids must be obtained from foods (meat, fish, eggs, and milk). Non-essential amino acids can be synthesized systemically. Essential amino acids, including histidine, isoleucine, leucine, lysine, methionine, phenylalanine, threonine, tryptophan, and valine are shown in the light red box, while non-essential amino acids, including alanine, arginine, asparagine, aspartic acid, cysteine, glutamic acid, glutamine, glycine, proline, serine, and tyrosine, are shown in the sky-blue box. Amino acid metabolites that are conditionally essential for systemic development and retinal protection, including taurine, are shown in the dark brown box. Amino acids are primarily used for protein synthesis, but when amino acids are plentiful, they are also used as metabolic fuel to support energy production. Ketogenic amino acids can form acetoacetate or acetyl-CoA. Glucogenic amino acids can be converted to pyruvate, alpha-ketoglutarate, succinyl-CoA, fumarate, or oxaloacetate. Leucine and lysine are only ketogenic amino acids. Phenylalanine, isoleucine, threonine, tryptophan, and tyrosine (five) are both ketogenic and glucogenic. The remaining amino acids are categorized as glucogenic amino acids.

More than 20 amino acids comprise the building blocks of proteins [1]. To date, about 500 amino acids have been identified in nature. Some amino acids needed for protein synthesis are “essential”, as they cannot be synthesized in sufficient quantities endogenously [2] and need to be provided exogenously. These include histidine, isoleucine, leucine, lysine, methionine, phenylalanine, threonine, tryptophan, and valine (Figure 1). These are available through dietary intake of animal proteins, including milk, meat, fish, eggs, and poultry. Other amino acids such as taurine are not constituents of proteins but are essential in other roles. Some plants (e.g., soy products) also contain essential amino acids. While non-essential amino acids (such as, alanine, arginine, asparagine, aspartic acid, cysteine, glutamic acid, glutamine, glycine, proline, serine, and tyrosine) can be synthesized from essential amino acids or from their metabolic intermediates, the building blocks could arise from glucose metabolism [3,4]. When the level of amino acids is sufficient to meet the demands of protein synthesis in the body, the excess amino acids could be metabolized to several compounds that are useful for energy production [5,6]. Ketogenic amino acids can be metabolized to acetoacetate or acetyl-CoA, while glucogenic amino acids can be metabolized to pyruvate, alpha-ketoglutarate, succinyl-CoA, fumarate, or oxaloacetate. Leucine and lysine are exclusively ketogenic. Five amino acids are amphibolic (which means both ketogenic and glucogenic): phenylalanine, isoleucine, threonine, tryptophan and tyrosine. The remaining amino acids are glucogenic.

Amino acid deficiency or imbalance can significantly result in systemic metabolic diseases [7,8,9,10,11,12,13,14]. Eye diseases might also be affected, including diabetic retinopathy and age-related macular degeneration [15,16,17]. The general pathology of eye diseases has been discussed in previous reviews [18,19] and includes retinal or choroidal neovascularization, inflammation, and cell death. These pathologic features are highly linked to each other. Supplementation with amino acid metabolites can influence retinal diseases, e.g., branched-chain amino acids metabolisms might be related to neuroprotection, oxidative stress, or pathologic inflammation in retinopathies [20]. Marine products containing high levels of amino acids (especially taurine and histidine) have therapeutic effects in retinal diseases [21]. Therefore, we reviewed clinical and experimental data concerning the potential of taurine and histidine supplementation to protect against eye diseases as well as supplementation with other amino acids (e.g., serine, arginyl-glutamine, and isoleucine).

## 2. Amino Acid Metabolism and Function

### 2.1. Taurine Metabolism and General Function

**Figure 2:**
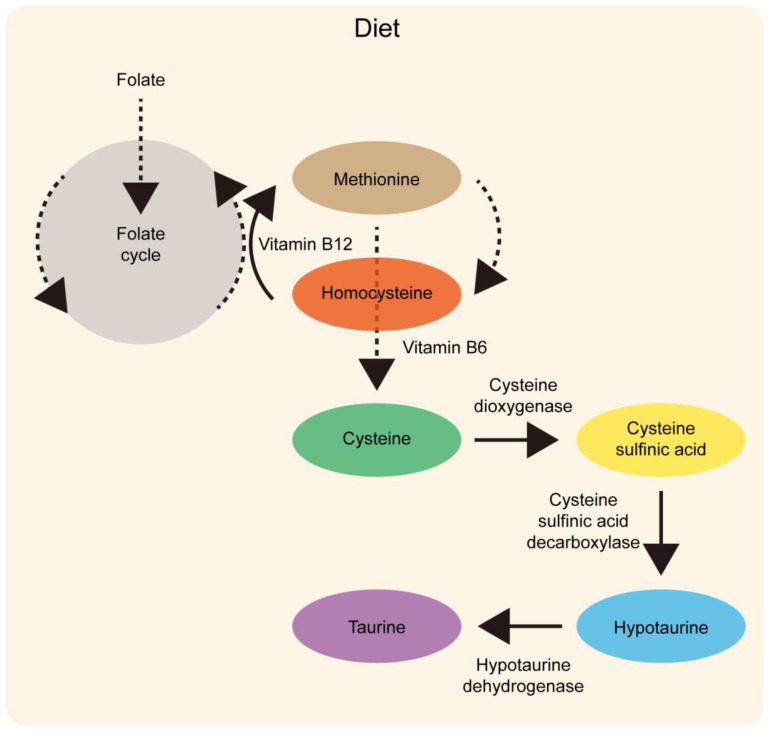
Taurine biosynthesis from several amino acids. Amino acids and vitamins are supplied from the diet. Homocysteine, an intermediate in conversion of methionine to cysteine, can be recycled and transformed into methionine by the enzyme methionine synthase in reactions with a series of folate cycles and vitamin B12. Taurine synthesis proceeds from cysteine (from methionine and homocysteine) to cysteine sulfinic acid, hypotaurine, and finally taurine with the involvement of cysteine dioxygenase, cysteine sulfinic acid decarboxylase, and hypotaurine dehydrogenase. The folate cycle (the folate-dependent one-carbon metabolism), which is important for development and survival, produces nicotinamide adenine dinucleotide phosphate (NADPH), as well as nucleotide precursors. Folate is mainly found in vegetables and nuts. Oranges, lemons, melons and strawberries are also rich in folate. The synthetic form of folate is folic acid. Folic acid supplementation is highly recommended during pregnancy. Cysteine has antioxidant properties and participates in redox reactions. Methionine might also act as an important endogenous antioxidant. Solid lines show a direct pathway. Dotted lines indicate an indirect pathway with several potential reaction steps.

Taurine (2-amino-ethane-sulfonic acid; C_2_H_7_NO_3_S) is a naturally occurring amino sulfonic acid. Unlike other amino acids, taurine is not incorporated into protein. Taurine can be obtained from the diet (especially mammalian milk, meat, fish, or energy drinks), and is also synthesized from amino acids, including cysteine (which is essential for antioxidant properties and redox reactions [22]) and methionine (which is important for the endogenous antioxidant defense system to protect cells [23,24,25]) (Figure 2). Taurine is one of the most abundant amino acid-derived products in the central nervous system, including the retina [26,27]. Other tissues, such as the heart, liver, or muscle, also contain a high level of taurine [28,29], which may modulate systemic physiological processes.

Supplementation with taurine during development can affect organ growth and general health in adulthood [30]. Sturman et al. found that adult female cats fed a taurine-free diet prior to breeding exhibited signs of reproductive dysfunction [31] as well as retinal degeneration, including photoreceptor outer segment loss. The maternal offspring had reduced systemic taurine levels and also had neurological abnormalities. In rhesus monkeys, Neuringer and Sturman found reduced visual acuity in taurine-deprived infants [32]. Imaki et al. reported similar findings. Taurine depletion induced retinal degeneration in infant rhesus monkeys [33]. Thus, taurine supplementation is essential during development, and is directly related to physiologic retinal function.

Heller-Stilb et al. disrupted the taurine transporter gene (*Taut*) in mice and found large reductions in systemic taurine levels (e.g., plasma, liver, kidney, heart, skeletal muscle, and eye), reduced fertility, and retinal degeneration (near-absence of nuclei in the outer retina and low electroretinography [ERG] a-wave and b-wave amplitudes) [34]. Kim et al. also showed that taurine depletion resulting from selective inhibition of its transporter can lead to impaired retinal functional in mice [35]. Thus, taurine and its transporter are necessary for physiologic retinal function and development.

The timing of taurine supplementation is likely to be important. Sturman et al. found that levels of taurine in rat milk are high for the first few days after birth and then rapidly decrease [36]. Using [35S] taurine as a tracer, they found that taurine is transferred from mothers to pups through milk [37]. High levels of taurine are present in both rat milk and human milk. Philipps et al. found that taurine levels are highest among the free amino acids/nutrients in the human placenta [38]. Maternal taurine is given continuously to offspring via the placenta or after birth through breast milk. As mammalian fetuses/infants are unable to effectively synthesize physiologic levels of taurine, maternally derived taurine might be necessary for proper fetal and infant development.

### 2.2. Therapeutic Effects of Taurine in Retinal Diseases

Taurine supplementation is one of the promising neuroprotective strategies in neurological [39,40] and retinal diseases. Froger et al. demonstrated that ingesting taurine through drinking water (0.2 M) provides dramatic protection against retinal ganglion cell damage in DBA/2J mice (a widely used genetic experimental glaucoma model), in rats with retinal vein occlusion, and in P23H rats (a model of retinitis pigmentosa with secondary retinal ganglion cell loss) [41]. Hadj-Saïd et al. suggested that a neuroprotective effect of taurine on retinal ganglion cells might be related to GABA_B_ receptor activation [42]. Jafri et al. found that taurine treatment protects against N-methyl-D-aspartate (NMDA)-induced rat retinal ganglion cell damage by modulating retinal oxidative stress control enzymes (e.g., glutathione, superoxide dismutase, and catalase) [43]. When taurine was intravitreally injected as pre-, co- or post-treatment with NMDA, pre-treatment was most protective against ganglion cell loss, suggesting that taurine supplementation could be used therapeutically to manage glaucoma [44,45].

Martínez-Vacas et al. found that taurine treatment administered via drinking water (0.2 M) protects photoreceptors, which have an increased number of photoreceptor outer segments, a reduced number of apoptotic cells in the outer retina, and increased ERG responses [46]. Taurine restored retinal pigment epithelium (RPE) function in dystrophic Royal College of Surgeons (RCS) rats with retinal degeneration secondary to impaired RPE phagocytosis caused by a MERTK mutation. Taurine supplementation increases plasma taurine levels and reduces oxidative stress. Martínez-Vacas et al. [47] found that taurine depletion causes slight shortening of photoreceptor outer segments and increases microglial activation and oxidative stress in the retina. In taurine-depleted animals, light-induced retinal damage significantly increased. As photoreceptor loss is a characteristic of retinal degenerative diseases, taurine supplementation might be beneficial for such conditions, including retinitis pigmentosa or dry age-related macular degeneration [48,49].

**Figure 3:**
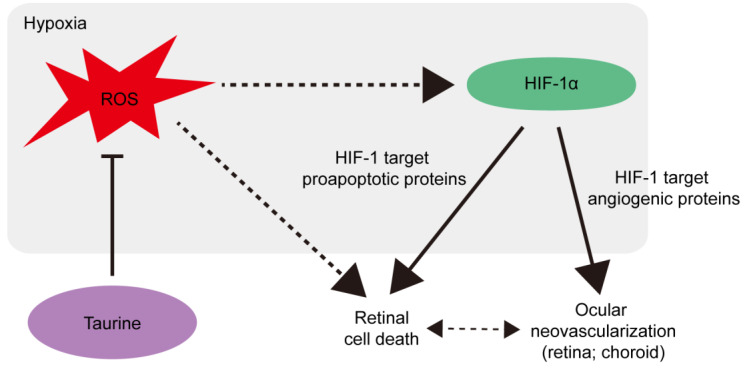
The possible mechanism of taurine-induced anti-angiogenic and anti-apoptotic effects in eye diseases. Oxidative stress plays an important role in retinal physiologic and pathologic metabolism. As retinal physiology is tightly linked with redox signaling, a balance between retinal reactive oxygen species (ROS; red) and antioxidant scavengers should be maintained. Hypoxic insults (represented by the gray box) could increase ROS levels in ocular cells (neural retina, retinal pigment epithelial cells, or choroid). Hypoxia-induced ROS might stabilize HIF or increase hypoxia-inducible factor-1 alpha (HIF-1α) expression in the eye, leading to increases in HIF-1 target angiogenic proteins (vascular endothelial growth factor (VEGF); placental growth factor (PGF); or erythropoietin (EPO)). The mechanism of ROS regulation of HIF-1α levels is in need of further investigation for each disease condition. One potential mechanism is that ROS may affect the HIF-1α proteasomal degradation pathway (especially functions of HIF regulatory protein prolyl hydroxylases (PHDs)). The pathologic process resulting from HIF-1α activation eventually causes neovascularization in the retina or choroid, eventually leading to visual impairments and vision loss. Retinal cell death is partly associated with HIF-1α-mediated apoptosis. Retinal cell death and neovascularization are also closely related. Damaged retinal vessels cause retinal inflammation and infiltrated inflammatory cells could affect retinal cell death or function. During the retinal cell death process, various chemokines or cytokines could be released and retinal glial cells activated, and the pathologic effects of these mechanisms could damage retinal vessels, ultimately contributing to the development of neovascularization. Either detrimental mechanism should be well understood in the eye (depending on the neural retina, retinal pigment epithelial cells, or choroid). Parts of the process could be suppressed by the antioxidant effects of taurine. Solid lines show direct effects. The dotted lines indicate indirect effects with several potential steps.

Pathologic angiogenesis and vascular dysfunction in the eye can lead to severe visual impairment [50,51]. Taurine supplementation (500 mg/kg) dramatically reduces retinal neovascularization [21] in a murine model of oxygen-induced retinopathy (OIR) [52], modeling retinopathy of prematurity and human ischemic retinopathies, including retinal neovascularization in diabetic retinopathy. Laser-induced choroidal neovascularization (which models neovascular age-related macular degeneration [53]) is also significantly suppressed as a result of taurine administration (0.5 g/kg) [21]. As a main mode of action, taurine inhibits pathologic hypoxia-inducible factor-1 alpha (HIF-1α) activation in ocular cells. HIF-1α is one of the important transcription factors functioning as an effective regulator of oxygen homeostasis and retinal physiologic and pathologic metabolism [54]. Preclinical studies as well as clinical data indicate that ocular ischemia-induced stabilization of retinal HIF-1α can cause retinal vascular and neuronal dysfunction, and vision loss in many sight-threating diseases including age-related macular degeneration, retinopathy of prematurity, diabetic retinopathy, and glaucoma [55,56,57,58]. Generally, HIF-1α can be activated by hypoxia or oxidative stress, further inducing hypoxia-responsive genes, such as vascular endothelial growth factor, platelet-derived growth factor, fibroblast growth factor, pyruvate dehydrogenase kinase, BCL2/adenovirus E1B 19 kDa protein-interacting protein, or glucose transporter [59,60,61,62,63]. These genes are associated with pathologic angiogenesis (also termed as neovascularization), inflammation, glucose metabolic regulation, or cell death, survival, and proliferation. Although taurine inhibition of HIF-1α activation in the eye under stress conditions still needs further investigation, hypoxia-induced reactive oxygen species regulation could be a therapeutic target (Figure 3), in that taurine is a strong antioxidant in different cell types [64].

In the OIR mouse retina modeling vessel loss and neovascularization, Zhou et al. identified (with metabolomics), the metabolic changes and their related pathways involved in ocular neovascularization [65]. Although they mainly focused on other metabolic factors and their relevant pathways in their article, dramatic taurine dynamics are seen in the datasets. We also detected the potential involvement of ocular taurine in ischemic retinopathy, although its direct role has not been mainly studied [66]. Harman et al. also conducted metabolomics in the mouse retina of hyperglycemia-associated retinopathy (HAR; modeling hyperglycemia-induced inhibition of normal vascular growth, Phase I retinopathy) [67]. They characterized the contributions of various amino acids in retinal vascular dysfunction in retinopathy of prematurity and diabetic retinopathy. Although the study mainly focused on three of the most suppressed amino acids, (L-leucine, L-isoleucine, and L-valine) in the metabolic profile from the retina with HAR, a dramatic change in taurine levels was observed in the retina with HAR. Furthermore, based on pathway analysis, taurine and hypotaurine metabolism has been found among the top 25 enriched metabolite sets. The role of taurine in retinal vascular dysfunction should be further examined as one of the potential therapeutic targets. With other accumulating experimental evidence together, taurine supplementation might be beneficial to reduce retinal injury in various ways (Table 1).

The beneficial effects of taurine have been studied clinically. Age-related systemic metabolic diseases (such as diabetes or cardiovascular diseases) [68], neurologic disorders, and several types of cancers have been targeted (ClinicalTrials.gov ID: NCT01226537, NCT00217165, NCT05149716, NCT04192539, NCT04874012, NCT04291352, and NCT06128252). Taurine has been reported to have anti-aging, antioxidant, and anti-inflammatory effects. However, clinical studies on taurine’s effects are needed so that both aspects (positive and negative) can be understood. Although retinal diseases have not been extensively examined [69], potentially positive effects of taurine supplementation have been identified based on the promising data from systemic metabolic diseases which are highly associated with retinopathy [70,71].

**Table 1:** Effects of taurine in experimental retinopathy.

Animal	Experimental Model	Dose/Method	Main Experimental Results	Reference
Rats and mice	(1) DBA/2J (2) Vein occlusion (3) P23H	0.2 M taurine in drinking water	Increases in retinal ganglion cell densities	[41]
Rats	Endothelin-1 (ET-1)-induced retinal and optic nerve injury	320 nM taurine administered intravitreally	Improvement in caspase activities and optic nerve morphology	[72]
Rats	NMDA-induced changes in retinal redox status and cell death	2 μL taurine intravitreally	Reduction in retinal oxidative stress and preservation in retinal cell density in inner retinas	[43]
Rats	Dystrophic Royal College of Surgeons (RCS)	0.2 M taurine in drinking water	Decreases in photoreceptor degeneration and increases in electroretinographic responses	[46]
Rats	Light-induced retinopathy	4% taurine in diet	Reduction in retinal damage by photochemical stress through antioxidant mechanisms	[73]
Rats	Streptozotocin-induced diabetic retinopathy	1.2% taurine in diet	Modulation in glial fibrillary acidic protein and glutamate transporter expressions	[74]
Mice	(1) Oxygen-induced retinopathy (2) Laser-induced choroidal neovascularization	500 mg/kg taurine in drinking water	Decreases in retinal and choroidal neovascularization	[21]

### 2.3. Histidine Metabolism and General Function

**Figure 4:**
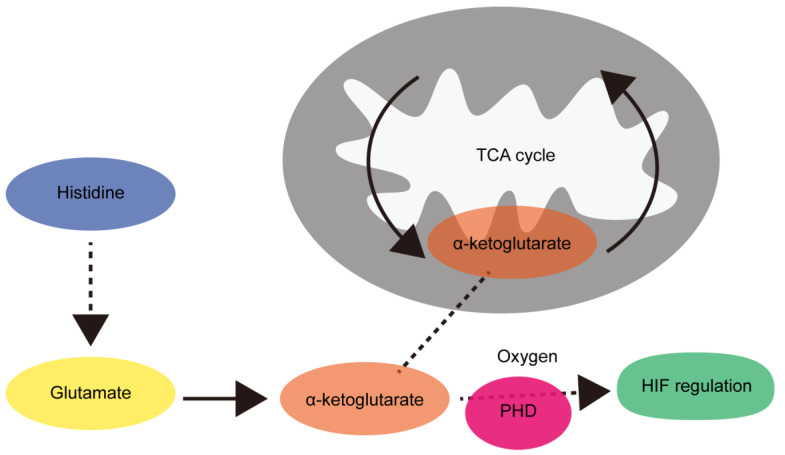
The histidine metabolic pathway may support energy metabolism and regulate HIF. Histidine can be converted to glutamate in four enzymatic steps. Glutamate can be converted to α-ketoglutarate by glutamate dehydrogenase. Alpha-ketoglutarate is one of the key intermediates in the tricarboxylic acid (TCA) cycle for ATP production. The TCA cycle (oxaloacetate → citrate → isocitrate → α-ketoglutarate → succinate → fumarate → malate → oxaloacetate) is located in the mitochondrial matrix (represented by the gray and white circle). A deficiency in activity in any enzyme in the TCA cycle could cause fundamental metabolic dysfunction. Oxygen and α-ketoglutarate are necessary for PHDs to allow HIF-1α proteasomal degradation. Glutamate is known to be the most abundant excitatory neurotransmitter in the central nervous system, which is critical for maintenance of energy levels in the brain, and is necessary for its physiologic functions and neuroplasticity [89]. Glutamate is also the dominant neurotransmitter of the retinal network, and its receptors play a pivotal role in retinal physiologic function.

Histidine is an essential amino acid and has important roles in buffering protons, chelating metal ions (such as Fe^2+^, Cu^2+^, Co^2+^, Ni^2+^, or Zn^2+^), and scavenging reactive oxygen and nitrogen species [75,76]. Histidine was first isolated in 1896 [77,78]. Histidine metabolism has focused on histamine, an important immune cell metabolite [79]. As histidine is present in meat, chicken, and fish, histidine deficiency is relatively rare. On the other hand, histidinemia, a rare autosomal recessive metabolic disorder caused by a deficiency of the enzyme histidase, is associated with an elevated level of histidine in the blood, urine, and the cerebrospinal fluid surrounding the brain and spinal cord [80,81,82]. Histidine can be converted to an intermediate in the tricarboxylic acid (TCA) cycle (Figure 4). The TCA cycle is a series of interrelated reactions forming a metabolic engine in cells, contributing to energy metabolism [83,84]. Briefly, this loop starts from oxaloacetate with acetyl-CoA and subsequently forms citrate. Citrate is further converted to isocitrate, which is decarboxylated to form alpha-ketoglutarate. Alpha-ketoglutarate undergoes decarboxylation to succinyl-CoA, which can be converted to succinate, fumarate, malate, and oxaloacetate. Then, oxaloacetate is used to regenerate the starting molecule and support the next round of the TCA cycle. In the electron transport chain, oxidative phosphorylation leads to ATP production. Balanced supplementation with histidine could enhance cellular energy metabolism.

Appropriate intake of histidine may improve metabolic syndrome related to obesity. In an internet-based cross-sectional study, Li et al. suggested that higher dietary histidine is inversely linked with energy intake, insulin resistance, and oxidative stress and inflammation in overweight/obese subjects in a northern Chinese population [85]. Kasaoka et al. also found that histidine affects food intake and fat accumulation in male Wistar rats [86]. Changes in food intake affecting levels of dietary histidine might be related to histamine activities in the brain [87,88], as histidine can be converted to histamine by histidine decarboxylase. This implies that histidine supplementation might help manage systemic metabolic diseases and neurological disorders.

### 2.4. Therapeutic Effects of Histidine in Retinal Diseases

While the neuroprotective role of histidine has been examined in central nervous system diseases (cerebral ischemia and epileptic seizures) [90,91], retinal diseases have not been directly examined. Nonetheless, based on metabolomics analyses, changes in histidine levels have been gradually reported in the plasma/serum and vitreous humor of subjects with diabetic retinopathy and age-related macular degeneration [92,93,94,95,96]. Although histidine has the potential to be used as a diagnostic biomarker in ocular disease states, the exact protective, preventive, or detrimental roles of histidine in diseases in the eye need to be unraveled.

The anti-angiogenic roles of histidine, along with marine natural products with high levels of histidine, have been evaluated in relation to eye diseases. Supplementation with histidine (5000 mg/kg) reduces retinal neovascularization in OIR mice and inhibits HIF-1α stabilization in ocular cells [21]. Torii et al. also found that histidine dramatically inhibits cobalt chloride-induced HIF-1α expression [97]. Jeong et al. found that HIF-1 stabilization with hypoxia regulates histidine decarboxylase expression [98]. As chronic HIF-1α stabilization in the eye can induce pathologic neovascularization as well as retinal dysfunction [99], supplementation with histidine could be assessed as a novel therapeutic strategy for managing retinopathy.

Investigations focusing on the beneficial effects of histidine in clinical studies have concentrated on neurologic disorders (ClinicalTrials.gov ID: NCT06169826 and NCT03266965) with no studies in ocular diseases. More preclinical evidence on the therapeutic roles of histidine in retinal diseases might be needed to move to the next research level.

### 2.5. Potential Role of Other Amino Acids in Retinal Physiology and Pathology

Although we focus on metabolism of taurine and histidine in the current review article, as their anti-angiogenic roles in the eye have been recently reported, other essential and non-essential amino acids are also important to retinal physiology and retinal disease development [100,101,102,103].

Niklaus et al. demonstrated that post-synaptic glutamate transporters directly regulate synaptic transmission in zebrafish retina [103]. Adler et al. suggested that glutamine might be the only amino acid that could adequately support NADPH generation in murine rod photoreceptors analyzed with fluorescence imaging of all-trans retinal and retinol [104]. Furthermore, they found that formic acid suppresses generation of NADPH.

Attallah et al. found that dietary supplementation with antioxidants and omega-3 long-chain polyunsaturated fatty acids promotes retinal glutamine synthesis in Müller glia [105] that might protect the retina under oxidative stress. Ardourel et al. suggested that glutamine synthesis might be essential to protect the retina against oxidative stress-induced retinal damage [106].

Neu et al. used the dipeptide arginyl-glutamine (Arg-Gln) to inhibit retinal neovascularization in OIR mice [107]. The inhibitory effect of Arg-Gln was associated with reduction in retinal *Vegf* mRNA expression. L-isoleucine administration physiologically delays normal retinal vessel growth in the hyperglycemic neonatal retina modeling Phase I ROP [67], indicating that supplementation with several amino acids might be useful to promote normal growth of retinal vasculature.

Low serine levels as well as disrupted serine biosynthesis have been associated with MacTel, a rare neovascular eye disease [108,109,110,111], and a clinical trial of serine supplementation is ongoing (ClinicalTrials.gov ID: NCT04907084). This Phase 2a study is evaluating the effects of serine supplementation and fenofibrate treatment (an FDA-approved peroxisome proliferator-activated receptor α-agonist/drug used to treat patients with hypertriglyceridemia, primary hypercholesterolemia, or mixed dyslipidemia) on elevated serum deoxysphingolipid levels in patients with MacTel.

Metabolomics (along with enrichment analyses) has enabled identification of metabolites [112], which might be involved in the pathogenesis of various retinopathies [113], as well as in normal physiology of ocular tissues [114,115,116]. These novel analytic methods uncover changes in the levels of many molecules, including glutamine, alanine, leucine, isoleucine, tyrosine, glutamate, valine, glycine, tryptophan, homocysteine, creatine, lysine, aspartate, or proline metabolism, that may or may not be critical in the pathophysiology of disease. To gain an in-depth understanding of the role of specific amino acids in retinopathies, supplementation, or deficiency studies of amino acids in experimental models of retinal diseases (e.g., OIR [retinopathy of prematurity], HAR [diabetic retinopathy], laser-induced choroidal neovascularization as well as subretinal fibrosis [wet age-related macular degeneration], light-induced retinopathy [dry age-related macular degeneration or retinitis pigmentosa], retinal ischemia/reperfusion injury [glaucoma], microbeads-mediated chronic intraocular pressure elevation-induced retinopathy [glaucoma], NMDA-induced retinal degeneration [glaucoma or diabetic retinopathy], carotid artery or other retinal vessel occlusion-induced ischemic retinopathy [various types of eye stroke], or streptozotocin-induced diabetic retinal injury [diabetic retinopathy]) are required. This will provide us with intriguing insights into the novel roles or effects of each amino acid in retinal physiology and pathology.

## 3. Future Directions and Conclusions

We summarize published evidence of the potential to treat eye diseases with supplementation of some amino acids, particularly taurine and histidine. However, although the relatively small number of studies conducted show encouraging results, more are needed to better define protective roles in disease states. Taurine has great potential as a neuroprotective (antioxidant) and anti-angiogenic agent to provide protection against retinal diseases. Histidine is also a potential amino acid-based therapeutic target for retinal protection. Future studies evaluating other essential and non-essential amino acids in disease conditions may uncover novel therapeutic targets for retinopathies. Optimized combinations of amino acids and other nutritional supplements should also be evaluated. Interestingly, Kim et al. demonstrated that a mixture of creatine (an organic compound with the nominal formula CNCH₂CO₂H) and taurine alleviates depressive behavior in *Drosophila melanogaster* and in mice, resulting in decreased levels of stress hormones and cytokines [117]. Combining caffeine with taurine enhances cognitive function and locomotor activities in sleep-deprived mice and modulates oxidative stress and inflammation [118].

Supplementing amino acids or other nutrients (such as vitamins or lipids) affect systemic disorders as well as the eye. Local injections of each amino acid could be potentially considered for further study with optimization of the injection dose and evaluation of adverse effects.

Systemic metabolic diseases are strongly linked to the progression of ocular diseases, such as diabetic retinopathy, central retinal artery occlusion, cataracts, age-related macular degeneration, glaucoma, pathologic myopia, and dry eyes [70,71]. Diabetic retinopathy is one of the most common microvascular complications in diabetic subjects, although other critical factors (e.g., hypertension or dyslipidemia) affect an individual’s susceptibility to its progression [119,120,121]. Although increased intraocular pressure is a major factor associated with the development of glaucoma, normal tension glaucoma cases also exist, suggesting possible systemic factors [122,123,124]. Intraocular pressure elevation is also affected by systemic cardiovascular conditions, insulin resistance, or obesity [125,126,127]. Therefore, a comprehensive understanding of the effects of amino acid supplementation in retinal diseases with systemic metabolic dysregulation is highly needed.

Globally, the energy drink industry is the fastest growing market. Energy drinks contain large amounts of caffeine, sugar or artificial sweetener, vitamins, taurine, and other ingredients. Energy drinks may have positive beneficial effects on exercise performance. However, there is a lack of evidence examining the potential side effects of energy drink consumption. Doğan et al. found that energy drink consumption leads to retinal vessel change [128]. Therefore, the possibility of potential detrimental health problems based on the biologic effects of each ingredient of energy drinks should not be overlooked locally (e.g., retinas) or systemically [129,130].

In conclusion, given the lack of promising preventive treatment of retinal pathologies in ocular diseases (such as diabetic retinopathy, central retinal artery occlusion, age-related macular degeneration, or glaucoma), administration of amino acids, either alone or in association with current ophthalmic drugs (especially anti-VEGF antibodies) or other promising nutrients (such as vitamins or minerals), could prevent retinal damage by improving systemic metabolic regulation. This review article suggests the potential therapeutic roles of amino acid supplementation in retinal diseases.
